# Can neuron modeling constrained by ultrafast imaging data extract the native function of ion channels?

**DOI:** 10.3389/fncom.2023.1192421

**Published:** 2023-05-24

**Authors:** Luiza Filipis, Marco Canepari

**Affiliations:** ^1^Univ Grenoble Alpes, CNRS, LIPhy, Grenoble, France; ^2^Laboratories of Excellence, Ion Channel Science and Therapeutics, Valbonne, France; ^3^Institut National de la Santé et Recherche Médicale, Paris, France

**Keywords:** neuron modeling, ion channels, ultrafast imaging, membrane potential, calcium, sodium

## Introduction

Since the discovery that the neuronal action potential is due to a sequential activation of voltage-dependent Na^+^ and K^+^ conductance (Hodgkin and Huxley, 1952a), Sir Alan Hodgkin and Sir Andrew Huxley proposed a set of differential equations (Hodgkin and Huxley, 1952b) that started modern computational neuroscience (Moore, 2010). Later, the cable theory adapted to neuronal compartments (Rall, 1962) allowed the first computer models of neurons (see, for example, Traub and Llinás, 1979 or Traub, 1982). Among the tools and languages that were developed in the last decades in order to efficiently simulate individual neuron behaviors, the NEURON programming language represents a critical step in the progress of computational neuroscience (Hines and Carnevale, 1997), but other languages such as GENESIS (Bower and Beeman, 2007) and NeuroML (Gleeson et al., 2010) are also used to perform virtual experiments. In parallel, the need of a neuron properties database was fulfilled by NeuronDB (Mirsky et al., 1998), which was followed by the model database ModelDB (McDougal et al., 2017). Thus, realistic models of neurons and synapses can be assembled to simulate large scale networks (Markram et al., 2015) or they can be used to inspire artificial networks (Chavlis and Poirazi, 2021). More realistic models of neurons can be further developed by constraining the parameters of the model to more detailed experimental datasets using optimisation procedures (Van Geit et al., 2008; Iavarone et al., 2019; Bologna et al., 2022). Following this direction, neuronal computational tools can be eventually used to derive biophysical models of native ion channels from experimental observations (Cannon and D'Alessandro, 2006). The optimisation of ion channel parameters, however, is typically performed using patch clamp recordings in voltage clamp mode. This approach is limited by the fact that the ionic current measured only at the site where the membrane potential (V_m_) is clamped is in general the summation of components originating from different cellular regions where V_m_ has different values (Williams and Mitchell, 2008). An alternative approach is to use ultrafast imaging recordings (at 50–200 μs time resolution) of physiological ion concentrations that are capable of resolving native Ca^2+^ currents (Jaafari et al., 2014; Jaafari and Canepari, 2016) or Na^+^ currents (Filipis and Canepari, 2021). In this case, ionic currents are measured at the site of origin where the variable physiological V_m_ can be measured, using voltage sensitive dyes, at the same time scale (Popovic et al., 2015). The contribution of individual channel types can be eventually estimated using highly selective inhibitors or boosters available for many ion channels, in particular molecules derived from toxins (Israel et al., 2018). This Opinion article is aimed at sharing our recent experience on using NEURON simulations to unravel the native behavior of ion channels from multiple ultrafast imaging recording in individual neurons in brain slices. This work not only allowed the measurement of ionic currents, but also extraction of the mutual functional interactions among diverse ion channels shaping the kinetics of physiological signals. Yet, the success of this approach is contingent not only upon the parameters of the model that can be experimentally assessed, but also upon those that cannot. Thus, we focus here on the critical aspects that must be addressed when working with novel rich experimental datasets that are becoming available from the use of state-of-the-art imaging techniques.

## The challenge of going from neurons to ion channels

**Figure 1:**
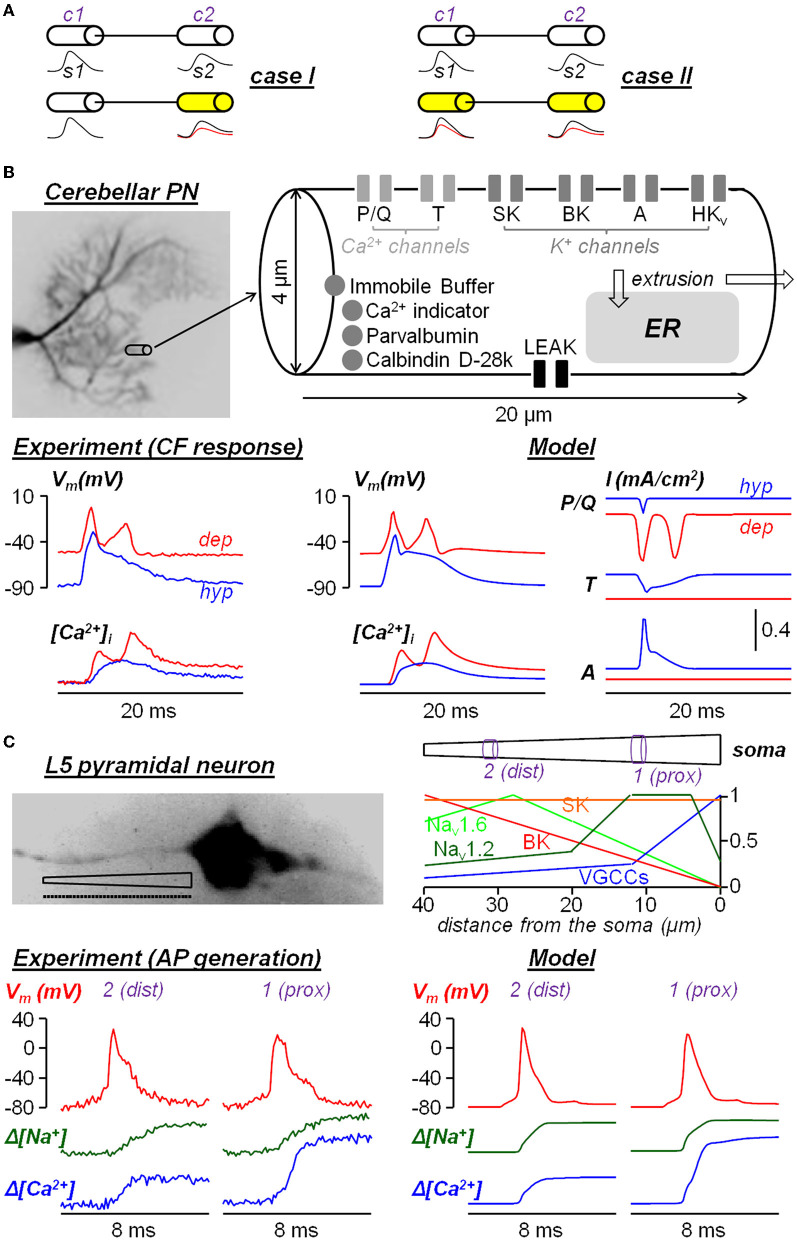
Two examples of neuron models built from experimental ultrafast ion and V_m_ imaging data. **(A)** Diagram of the two cases where a signal *s1* from compartment *c1* is transmitted to compartment *c2* to generate a signal *s1*. In *case I*, only *s2* changes after a pharmacological manipulation; in *case II*, both *s1* and *s2* change after a pharmacological manipulation. **(B)** Single-compartment model of dendritic response to climbing fiber (CF) stimulation in cerebellar Purkinje neurons reported in Ait Ouares et al. (2019). The model reproduces the dendritic V_m_ and Ca^2+^ transients associated with CF activation when the initial V_m_ was hold at hyperpolarised state (*hyp*, blue traces) or at depolarised state (*dep*, red traces). The currents of P/Q-type VGCCs, T-type VGCCs and A-type VGKCs in the two states are obtained from the computer stimulations. **(C)** Multi-compartment model (40 compartments of 1 μm length each, dotted line) of AP generation in the AIS of a L5 pyramidal neuron reported in Filipis et al. (2023). The model, where critical ion channels are unevenly distributed along the AIS, reproduces the V_m_, Na^+^ and Ca^2+^ transients during AP generation at a proximal site (*1, prox*) and at a distal site (*2, dist*) of the AIS.

The process of building realistic neuron models starts always from two sets of experimental information: (1) the knowledge of which ion channels and other molecules (ion pumps, buffers, etc.) are involved in the measured signals; and (2) a collection of physiological V_m_ signals which is sufficiently detailed to constrain the parameters of the model. For ion channels, the biophysical behavior of the model can be initially established from experiments where the channel is expressed in a host system. Each channel can be modeled as deterministic (Petousakis et al., 2022) or stochastic (Goldwyn et al., 2011) Hodgkin-Huxley function. From this background, the modeler can optimize the parameters of ion channels and of the other molecules so that the results of the computer simulation match the set of experimental V_m_ signals. When using electrodes, however, the consistency between experiments and simulations is limited to the site(s) of the electrode(s), whereas V_m_ imaging can significantly reduce this limitation allowing assessing multi-compartment models. In addition, ultrafast Ca^2+^ or Na^+^ imaging can provide direct evidence on the activation of Ca^2+^ or Na^+^ channels, respectively. Finally, the use of selective inhibitors or boosters of individual ion channel types allows direct assessment of the targeted channel. The accuracy of a neuron model can be therefore linked to the number of different experimental tests used to constrain it. Typically, a model based on ultrafast V_m_ and ion imaging recordings can be built starting from an existing model developed on electrodes measurements, where original and possibly additional parameters are further optimized on the richest set of experimental evidence. The result of this procedure is a more accurate model that not only reproduces the physiological V_m_ signals, but also the individual ionic currents of each channel type as well as the synergistic interactions of ion channels. To achieve a realistic ion channel function, however, some technical challenges must be taken into consideration to establish whether the approach may or may not work in each specific case. In general, when constraining the model on a cellular compartment, one should distinguish the case in which a constant signal is injected into a compartment, and the more common case in which the input itself is a variable (Figure 1A). In the first case, which is normally a simplified approximation, the input signal from the first compartment regulates the conductance producing an output signal in the second compartment, but the input is not affected by a pharmacological manipulation. In the second case, the input signal generated in the first compartment is also affected by the pharmacological manipulation. Clearly, whereas in the first case the optimisation can be performed at the level of a single compartment, in the second case it must be performed at the level of the multi-compartment system. The two following examples from our recent research, where automated optimisation algorithms were not used, are representative of the two different cases.

## Two examples from recent research

An example of simplified single compartment model (case *I* in Figure 1A), which was capable of extracting the behavior of native ion channels, was obtained for the dendrites of the cerebellar Purkinje neuron (PN) (Ait Ouares et al., 2019). As shown in Figure 1B, a dendritic segment of the size from which V_m_ and Ca^2+^ signals could be obtained at 200 μs temporal resolution with high signal-to-noise ratio (SNR). The segment was modeled as single compartment with 6 types of ion channels and various Ca^2+^ sequestration mechanisms, starting from an existing model available in the literature (Anwar et al., 2012). V_m_ and Ca^2+^ transients elicited by the climbing fiber (CF) stimulation were recorded at two different states: first by injecting current through a somatic patch clamp pipette to hold the initial V_m_ at hyperpolarised (*hyp*) state and then at depolarised (*dep*) state. Then, 5 of the 6 channels in the model (one at the time) were inhibited by local application of selective blockers. This way, in agreement with the assumption that the CF V_m_ input originating near the soma was not affected, the parameters of the ion channels were constrained by the dataset that included the blockade of individual channels. The result was a realistic reconstruction of the underlying ionic currents, in particular of the Ca^2+^ current mediated by T-type channels and of the K^+^ current mediated by A-type channels, that prevent the full activation of P/Q-type Ca^2+^ channels at hyperpolarised state, but that are inactivated at depolarised state allowing the generation of dendritic Ca^2+^ spikes mediated by P/Q-type Ca^2+^ channels. An example of multi-compartment model (case *II* in Figure 1A) was obtained from the study of the generation of the AP in the axon initial segment (AIS) of the neocortical layer-5 (L5) pyramidal neuron (Filipis et al., 2023). As shown in Figure 1C, the 40-μm long AIS was divided into 40 compartments with non-uniform ion channel distribution and the model was built from an existing model available in the literature (Hallermann et al., 2012). V_m_, Na^+^, and Ca^2+^ transients associated with the AP, elicited by the injection of a somatic current pulse, were recorded at 100 μs temporal resolution along the AIS. A fraction of Na_v_1.2 Na^+^ channels was inhibited using a partially selective blocker and other full blockers were utilized to test diverse voltage-gated Ca^2+^ channels and SK and BK Ca^2+^-activated K^+^ channels. Since signals with sufficient signal-to-noise ratio could be obtained only by averaging fluorescence over 5 μm long sites, the parameters of the ion channels (including their spatial distribution) were constrained on a distal (*dist*) site and on a proximal (*prox*) site of the channels only. The model could reproduce the V_m_, Na^+^, and Ca^2+^ transients unraveling the functional interaction between Na_v_1.2 and BK channels, but given the multi-compartmental nature of the problem, the estimate of the native ion currents in each compartment was less accurate than in the first example.

## Discussion

The two examples analyzed above represent a promising beginning toward the use of computational tools to extrapolate native physiological ionic currents from ultrafast V_m_, Na^+^, and Ca^2+^ recordings. This is crucial because the same ion channel can behave in a different manner in two distinct native contexts where it physically interacts with other molecules and only a measurement during a physiological signal can be informative of its realistic behavior. In the first system that we analyzed, where the problem was addressed with a single compartment model, the accuracy of the ionic currents obtained from computer stimulation was remarkably high. From our experience, we suggest a possible pathway necessary to improve this approach. On the experimental side, V_m_ and ion transients can be obtained from more compartments and with more pharmacological assessments of individual channels. On the computational side, the use of sophisticated optimisation procedures to build models from multiple experimental data (Nandi et al., 2022) can be combined with stepwise neuron model fitting procedures (Mäki-Marttunen et al., 2018) to exploit this novel experimental information. Our opinion is that this is a worth-doing effort because of its enormous potential impact on the understanding of functional ion channel organization and potentially of dysfunctions caused by channelopathies.

## Author contributions

MC wrote the manuscript and prepared the figure. LF extensively revised the manuscript. All authors contributed to the article and approved the submitted version.
